# PET Imaging of Neuroinflammation in Alzheimer’s Disease

**DOI:** 10.3389/fimmu.2021.739130

**Published:** 2021-09-16

**Authors:** Rong Zhou, Bin Ji, Yanyan Kong, Limei Qin, Wuwei Ren, Yihui Guan, Ruiqing Ni

**Affiliations:** ^1^Department of Nephrology, Yangpu Hospital, School of Medicine, Tongji University, Shanghai, China; ^2^Department of Radiopharmacy and Molecular Imaging, School of Pharmacy, Fudan University, Shanghai, China; ^3^Positron Emission Tomography (PET) Center, Huashan Hospital, Fudan University, Shanghai, China; ^4^Inner Mongolia Baicaotang Qin Chinese Mongolia Hospital, Hohhot, China; ^5^School of Information Science and Technology, Shanghaitech University, Shanghai, China; ^6^Institute for Regenerative Medicine, University of Zurich, Zurich, Switzerland; ^7^Institute for Biomedical Engineering, University of Zurich & Eidgenössische Technische Hochschule Zürich (ETH Zurich), Zurich, Switzerland

**Keywords:** Alzheimer’s disease, neuroinflammation, tau, microglia, astrocyte, amyloid (A) 42, positron emission tomography (PET), TSPO (18 kDa translocator protein)

## Abstract

Neuroinflammation play an important role in Alzheimer’s disease pathogenesis. Advances in molecular imaging using positron emission tomography have provided insights into the time course of neuroinflammation and its relation with Alzheimer’s disease central pathologies in patients and in animal disease models. Recent single-cell sequencing and transcriptomics indicate dynamic disease-associated microglia and astrocyte profiles in Alzheimer’s disease. Mitochondrial 18-kDa translocator protein is the most widely investigated target for neuroinflammation imaging. New generation of translocator protein tracers with improved performance have been developed and evaluated along with tau and amyloid imaging for assessing the disease progression in Alzheimer’s disease continuum. Given that translocator protein is not exclusively expressed in glia, alternative targets are under rapid development, such as monoamine oxidase B, matrix metalloproteinases, colony-stimulating factor 1 receptor, imidazoline-2 binding sites, cyclooxygenase, cannabinoid-2 receptor, purinergic P2X7 receptor, P2Y12 receptor, the fractalkine receptor, triggering receptor expressed on myeloid cells 2, and receptor for advanced glycation end products. Promising targets should demonstrate a higher specificity for cellular locations with exclusive expression in microglia or astrocyte and activation status (pro- or anti-inflammatory) with highly specific ligand to enable *in vivo* brain imaging. In this review, we summarised recent advances in the development of neuroinflammation imaging tracers and provided an outlook for promising targets in the future.

## Introduction

Neurodegenerative diseases, including Alzheimer’s disease (AD), frontotemporal dementia, Parkinson’s disease (PD), and Lewy body dementia, represent a tremendous unmet clinical need. The major neuropathological features of AD are the deposition of amyloid-beta (Aβ) plaques, neurofibrillary tangles formed by misfolded hyperphosphorylated tau, neuronal loss, and neuroinflammation characterised by glial activation (1, 2). Neuroinflammation plays an important role in AD; however, its dynamics and impacts (protective or detrimental) have still not been fully elucidated (3, 4). Microglia, as the resident macrophage cells in the brain, have emerged as central players in the AD pathogenesis (1, 2, 5). Microglial activation was previous classified into proinflammatory (M1) or anti-inflammatory (M2) types (2). Recent single-cell sequencing and transcriptomics studies reported gene coexpression network diversity of microglia in AD and disease-associated-microglia (DAM) of transcriptionally distinct and neurodegeneration-specific profiles (6–12). Aβ-laden microglia has a unique gene-expression signature including triggering receptor expressed on myeloid cells 2 (TREM2), apolipoprotein E (ApoE), and other AD-associated genes (13, 14). Microglia phagocytosis driven by Tyro3, Axl, and Mer (TAM) receptor has been shown to promote the development of dense-core plaque and the engulfing of Aβ plaques (15). Astrocytes are categorised into A1 and A2 subtypes based on their phenotype and genetic expression profiles (16–19). A1 astrocyte secretes and produces a large number of inflammatory factors and neurotoxins, whereas A2 astrocyte produces neurotrophic substances and supports neuronal growth. Reactive astrocytes precipitate both Aβ and tau (20–22) and are closely linked with microgliosis (16). Cerebrospinal fluid (CSF) and plasma biomarkers for neurodegeneration and inflammatory markers [e.g., tumor necrosis factor alpha (TNFα), interleukin-6 (IL-6), IL-10] were elevated in patients with AD and mild cognitive impairment (MCI) compared to healthy controls (6, 7, 13, 14), associated with an increasing age (23, 24) and cerebral amyloid pathology (25). Recent advances in molecular imaging have provided insights into the time course of AD pathology, including Aβ, tau, synaptic deficits, and neuroinflammation, in patients and in animal disease models (1, 26–35). *In vivo* imaging of neuroinflammation, however, is challenging, and the spatial–temporal pattern in the development of AD has still not been fully elucidated (23). One reason is that the astrocytes and microglia are highly dynamic and heterogeneous in their subtypes, locations, and activation status (1).

## Neuroinflammation Positron Emission Tomography Imaging

Mitochondrial 18 kDa translocator protein (TSPO) is the most widely investigated neuroinflammation target for PET imaging (36). Other alternative targets are under rapid development (**Table 1**), such as monoamine oxidase-B (MAO-B), matrix metalloproteinases (144–147, 185, 186), colony-stimulating factor 1 receptor (CSF1R), imidazoline-2 binding sites (I_2_BS), cyclooxygenases, the phospholipase A2/arachidonic acid pathway, sphingosine-1-phosphate receptor-1, reactive oxygen species, cannabinoid-2 receptor, purinergic P2X7 receptor and P2Y12 receptor, the fractalkine receptor (CX3CR1) (187), TREM2 (140), and receptor for advanced glycation end products (36, 188) (**Table 1**).

**Table 1:** Summary of imaging probes for gliosis.

Target	Tracer	Human	Animal model
TSPO	(R)-[^11^C] PK11195	MCI, AD, HC (37–43)	3×Tg, APP/PS1 mice, rTg4510 mice (26, 44)
	[^18^F]DPA-714	AD, MS, ALS, HC (45, 46)	APP/PS1 mice, TgF344 rats (47–51)
	[^11^C]DPA-713	AD, HC (52)	Murine stroke models (53), aged Monkeys (54)
	[^18^F]F-DPA		APP/PS1 mice (55)
	[^18^F]FEBMP		PS19, rTg4510 mice (56–58)
	[^11^C]DAA1106, [^18^F]FEDAA1106	AD, HC	APP23, APP/PS1, PS19 mice, TgF334 rats (50, 59, 60)
	[^18^F]FEMPA	AD, HC (61, 62)	
	[^11^C]AC-5216	HC (63)	APP23, App^NL-G-F/NL-G-F^-knock-in, APPE693, rTg4510, PS19 mice (26, 57, 60, 64, 65)
	[^18^F]FEPPA	MCI, AD, HC (66, 67)	TgAPP21 rats (68)
	[^11^C]PBR06	MCI, AD, HC (69–71)	APP^L/S^ mice (57, 72, 73)
	[^11^C]PBR28	AD, SD, MCI, FTD, DLB, ALS, HC (40, 69, 74–82)	5×FAD, PS19 mice (57, 73)
	[^18^F]PBR111		APP/PS1 mice (83, 84)
	[^125^I]CLINDE	AD, HC (85)	LPS injected, 3×Tg mice, TgF344 rats (85–87)
	[^18^F]GE-180	AD, MS, FTD, HC (88–91)	APP/PS1, PS2APP, APP23, APP-SL70, APPswe, APP^NL-G-F^, APP^L/S,^ Trem2 p.T66M knock-in, PS19 mice, TgF344 rats (47, 50, 53, 90, 92–102)
	(S)-[^18^F]GE-387, (R, S)-[^18^F]GE-387	HC (103)	LPS injected rats, non-human primates (103, 104)
	[^11^C]ER176	HC (75, 105, 106)	
	[^11^C]CB184, [^11^C]CB190		Mice, 6-OHDA injected rats (107)
	[^11^C]N′-MPB		Stroke rat model (108)
	[^18^F]LW223	HC (109)	Rats (109)
P2X7R	[^11^C]GSK1482160	HC (110)	LPS-injected mice, EAE rats, non-human primates (110, 111)
	[^18^F]JNJ-64413739	HC (112), ALS (113)	LPS injected mice (112, 114)
	[^11^C]JNJ-54173717	ALS (113)	rAAV3flag-hP2X7R, α-synuclein, 6-OHDA injected rats, non-human primates (115, 116).
	[^11^C]SMW139	MS (117)	EAE, rAAV3flag-hP2X7R rats (118, 119)
	[^11^C]JNJ-47965567 (A-740003)	MS (120)	Rats (121)
P2Y12R	[^11^C]P2Y12R-ant	MS (120)	EAE rats (120)
	[^11^C]5	Stroke (122)	Murine stroke model (122)
	[^11^C]AZD1283		rTg4510, PS19, APP23, and APP^NL-F/NL-F^ mice*, ex vivo* (123)
CSF1R	[^11^C]CPPC	AD, HC (124)	LPS injected, EAE, APPsi, APP^NL-G-F/NL-G-F^knock-in mice (64, 124)
	[^11^C]GW2580		LPS injected, APP^NL-G-F/NL-G-F^knock-in mice, non-human primates (64)
COX-1	[^11^C]-KTP-Me	AD, HC (125, 126)	APPswe mice (125–127)
	[^11^C]PS13, [^18^F]PS2		LPS treated rhesus macaques (128)
COX-2	[^11^C]MC1		LPS treated rhesus macaques (129)
	[^18^F]FMTP		LPS injected mice (130)
	[^18^F]TMI		Non-human primates (131)
iNOS	[^18^F]FBAT		LPS injected mice (132)
ROS	[^18^F]ROStrace [^18^F]ox-ROStrace,		LPS-treated mice (133)
	[^18^F]dihydromethidine,		LPS-treated mice (134)
	[^11^C]Ascorbic. [^11^C]dehydroascorbic acid		Rats (135)
	[^62^Cu]ATSM	PD, ALS, MELAS (136, 137)	Brain tumor mice (138, 139)
TREM-2	[^124^I]mAb1729,mAb1729-scFv8D3CL		APPArcSwe, APPswe mice (140)
TREM-1	[^64^Cu]TREM1-mAb		Murine stroke, MS, GBM models (141–143)
			Murine glioma and metastatic breast cancer model (144–146)
MMP	[^18^F]BR-351, [^18^F]BR-420		Murine stroke models (147, 148)
CB_2_R	[^11^C]A-836339, [^18^F]2f	HC (149)	J20APPswe/ind, APP/PS1 mice (150)
	[^18^F]RS-126, [^18^F]RoSMA-18-d6		LPS injected, Huntington, stroke mice (151, 152)
	[^18^F]JHU94620		LPS injected mice (153)
	[^11^C]NE40	AD, HC (154)	SAMP10 mice (155)
MAO-B	[^11^C]DED	MCI, AD, HC (156–162)	APPArcSwe, APPswe mice (163, 164)
	[^18^F]fluorodeprenyl-D2		Non-human primates (165)
	[^18^F]SMBT-1	AD, HC (166, 167)	
	[^11^C]SL25.1188	HC, MDD (168, 169)	LPS-injected rats (170)
astrocyte	[^11^C]acetate	MCI, MS, HC (171, 172)	
I_2_BS	[^11^C]BU99008	AD, PD, HC (173–178)	Zucker rats, Non-human primates (179–181)
	[¹⁸F]FEBU (BU99018)		Mice and rats (182)
	[¹¹C]FTIMD		Non-human primates (183)
OATP1C1	[^18^F]2B-SRF101		3×Tg mice (184)

ALS, amyotrophic lateral sclerosis; COX-1/2, cyclooxygenase 1/2; CSF1R, colony stimulating factor 1 receptor; DED, deuterium-L-deprenyl; FTD, Frontotemporal dementia; GBM, glioblastoma; HC, healthy control; iNOS, inducible nitric oxide synthase; I2BS, I2-imidazoline binding sites; LPS, lipopolysaccharides; MCI, mild cognitive impairment; MDD, major depressive disorder; MELAS, mitochondrial myopathy, encephalopathy, lactic acidosis and stroke-like episodes; MMP, matrix metalloproteinases; MS, multiple sclerosis; OATP1C1, organic anion-transporting polypeptide 1C1; ROS, reactive oxygen species; PD, Parkinson’s disease; SD, semantic dementia; TREM-1, 2, triggering receptor expressed on myeloid cells 1, 2; TSPO, translocator protein; 6-OHD, 6-hydroxydopamine.

### TSPO Imaging

**Figure 1:**
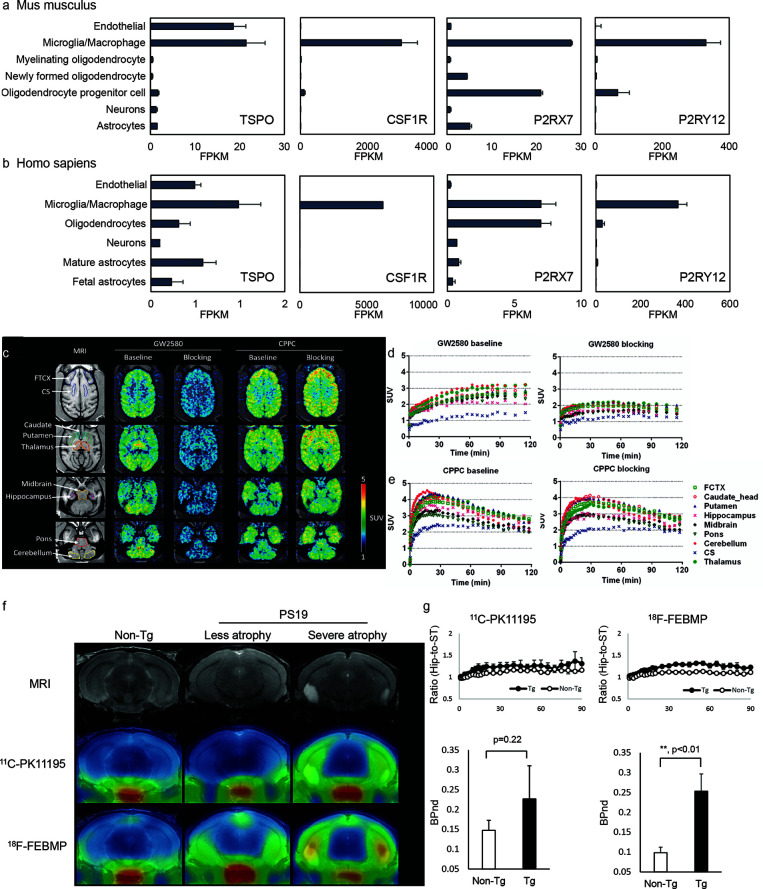
Cellular location of emerging neuroinflammation imaging targets. **(A, B)** The RNA expression of *TSPO, CSF1R, P2RX7*, and *P2RY12* in mouse **(A)** and human **(B)** brain [based on RNA-Seq data (189, 190)]. FPKM, fragments per kilobase of transcript per million mapped reads. Reproduced from https://www.brainrnaseq.org and (189, 190) with permission. **(C)** Representative transverse planes of [^11^C]GW2580 and [^11^C]CPPC SUV 60-120min images of a monkey brain superimposed on the monkey’s own MR images at baseline and with a homologous blocker treatment. **(D, E)** Time–radioactivity curves of [^11^C]GW2580 and [^11^C]CPPC in various brain regions obtained from corresponding PET images. FCTX, frontal cortex; CS, centrium semi-ovale. Reproduced from (64) with permission from Sage Publication. **(F, G)** Tau lesion-associated microglial TSPO was more sensitively captured by *in vivo* positron emission tomography (PET) imaging with [^18^F]FEBMP than [^11^C]PK11195. T2 magnetic resonance imaging (MRI) images and PET images with [^18^F]FEBMP and [^11^C]PK11195 in non-transgenic, and PS19 mice with less and severe brain atrophy at 9 months of age **(F)**. Time course of hippocampus (Hip)-to-striatum (ST) ratios of radioactivity and binding potential (BPnd) calculated by simplified reference tissue model with striatum as reference tissue showing significantly increased [^18^F]FEBMP but not [^11^C]PK11195 signal in PS19 compared with non-transgenic mice **(G)**. Reproduced from (57) with permission from Sage Publication.

TSPO is expressed mainly in the outer mitochondrial membrane of steroid-synthesizing cells in the central nervous system (microglia, astrocytes, endothelial cell, etc.) (**Figures 1A, B** **)** and in the peripheral (191). TSPO is involved in many physiological processes including transporting cholesterol into mitochondria, steroid hormone synthesis, and bioenergetics (191, 192). Upregulation of TSPO was found in patients with AD and in animal models of AD (92, 193).

#### The First Generation TSPO Tracers

The first-generation tracers exemplified with [^11^C]PK-11195 have been widely used in preclinical and clinical studies. However, [^11^C]PK-11195 suffers from several major limitations such as low permeability of the blood–brain barrier and high non-specific plasma binding, leading to a low signal-to-noise ratio in the final reconstructed PET images (194). Careful analysis of plasma metabolites is required to determine the accurate arterial input function for quantitative PET measurement (195). Increased [^11^C]PK11195 is reported to be associated with Aβ accumulation in patients with MCI and AD compared to healthy controls, correlating with the deficits in functional network connectivity, grey matters atrophy, and cognitive decline (37–39, 196). Using [^11^C]PK11195, recent studies have showed a biphasic trajectory of inflammation with an early microglial activation with increasing Aβ load and a later decline when Aβ load reaching plateau (AD) levels (40). Ismail et al. demonstrated a parallel increase in microglial activation and tau accumulation assessed by [^11^C]PK11195 and [^18^F]flortaucipir, respectively, in [^11^C]PIB Aβ-positive MCI patients (41). Su et al. further showed that grey matter atrophy mediated the effects of tau accumulation and neuroinflammation detected by PET tracers [^18^F]flortaucipir and [^11^C]PK11195, respectively on cognitive impairments in AD (42).

#### The Second Generation TSPO Tracers

**Figure 2:**
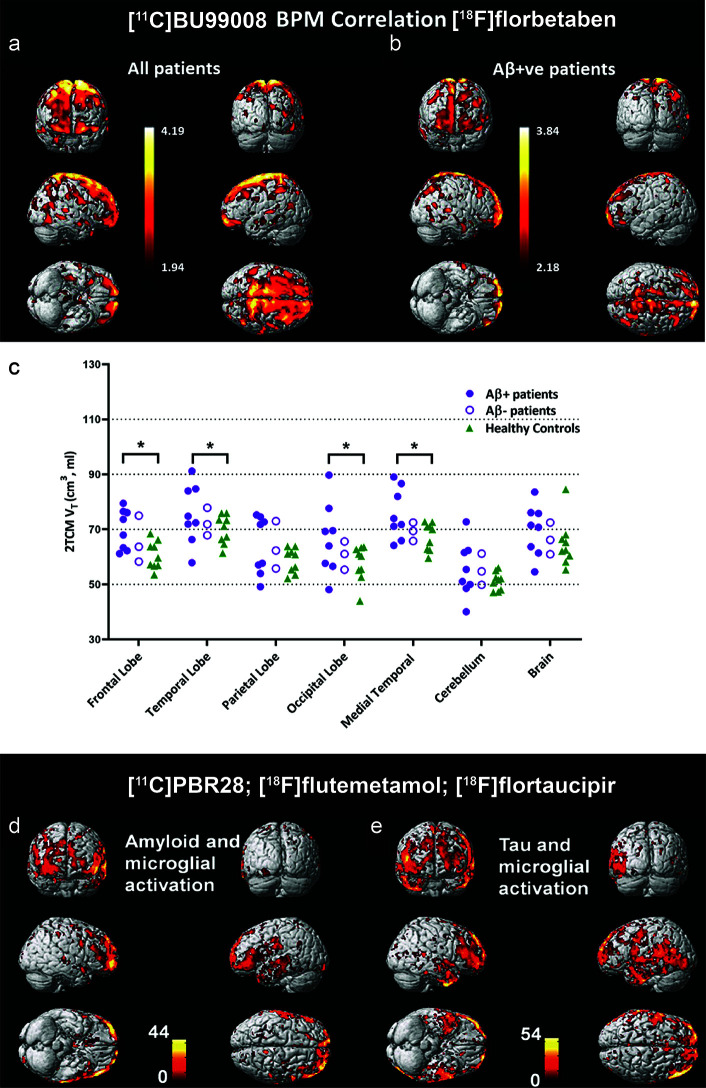
Biological parametric mapping (BPM) correlation between [^11^C]BU99008 and [^18^F]florbetaben binding in **(A)** all cognitively impaired (CI) subjects and in **(B)** Aβ-positive cognitively impaired subjects at a cluster threshold of *p* < 0.05 with an extent threshold of 50 voxels. These BPM are T maps describing the strength of the voxel-wise correlations between binding of the two radioligands represented in a common brain space. **(C)** Dot plot demonstrating the regional [^11^C]BU99008 total volumes of distribution (Vt) using two-tissue compartmental models in Aβ-positive cognitively impaired subjects (purple filled circle), Aβ-negative CI subjects (purple open circle), and healthy controls (HC, green triangle). “Brain” refers to the composite cortex, combining all the major cortical regions. *p < 0.05, uncorrected. Reproduced from (176) with permission from Springer Nature. **(D, E)** Voxel-level correlation between [^18^C]PBR28, [^18^F]florbetapir, and [^18^F]flutemetamol in the patients with mild cognitive impairment and Alzheimer’s disease who were positive for all three tracers. **(D)** Voxel-level correlations between microglial activation assessed by using [^18^C]PBR28 and tau aggregation assessed by using [^18^F]florbetapir. **(E)** Voxel-level correlations between microglial activation assessed by using [^18^C]PBR28 and amyloid deposition assessed by using [^18^F]flutemetamol. Reproduced from (69) with permission from Oxford University Press.

A few second generation tracers including [^11^C]DAA1106, [^1(^F]FEDAA1106, [^125^I]CLINDE [^11^C]PBR06, [^11^C]PBR28, [^18^F]PBR111, [^18^F]DPA-713, [^18^F]DPA-714, [^18^F]F-DPA, [^11^C]AC-5216, [^18^F]FEMPA, and [^18^F]FEPPA have been developed to overcome the limitations of [^11^C]PK11195 (45, 46, 52, 61–63, 66, 69–71, 83, 84, 197) (**Table 1**). However, the binding affinities of second generation TSPO tracers in human brain differ based on the *rs6971* polymorphisms, which introduces higher variability between subjects (45, 46, 52, 61–63, 66, 69–71, 197). In addition, the [^11^C]PBR28 binding appears to be affected by chromosome 1 variant *rs2997325* on microglial activation (198). Several longitudinal studies using [^18^F]DPA-714, [^11^C]DAA1106, and [^11^C]PBR28 have reported decreased glucose metabolism and increased neuroinflammation in amyloidosis, four-repeat tauopathy animal models (47–50, 55) (**Table 1**). Ishikawa et al. has indicated an association between tau assessed by [^11^C]PBB3, neuronal damage measured by structural MRI, and neuroinflammation detected by using [^11^C]AC-5216 in rTg4510 mice (56–58). Chaney et al. showed an increased [^18^F]DPA-714 binding and myo-inositol levels using ^1^H magnetic resonance spectroscopy in APP/PS1 mice (48). Zou et al. showed that microglial activation assessed by [^11^C]PBR28 is independently associated with amyloid load and memory impairment, but not with tau burden assessed by [^18^F]florbetaben and [^18^F]MK-6240, respectively, in patients with AD (74). Whereas Dani et al. showed that [^11^C]PBR28-measured microglial activation correlates with both tau and Aβ deposition assessed by [^18^F]flortaucipir and [^18^F]flutemetamol in patients with AD (69) (**Figures 2D, E**). Studies by Femminell et al. demonstrated an increased regional [^11^C]PBR28 binding in patient with MCI, which associated with higher grey matter and hippocampal volume (199). This suggests a potential protective effect of microglia activation in the early stages (199). Hamelin et al. showed a diverging pattern of progression in AD based on [^18^F]DPA-714 baseline binding, with a higher baseline associates with less subsequent microglial activation and better cognitive performance in 2-years follow-up (45).

#### The Third Generation TSPO Tracers

Several third generations of TSPO tracers [^18^F]GE-180, (R,S)-[^18^F]GE-387, [^11^C]ER176, [^11^C]CB184, [^11^C]CB190, [^11^C]N′-MPB, and [^18^F]LW223 have been developed (75, 103, 105–109). [^18^F]GE-180 (flutriciclamide), (S)-[^18^F]GE-387, and [^11^C]ER176 resolve the problem of ligand-dependent attenuation of affinity (90, 97, 197) in *in vitro* binding assay where these tracers are insensitive to TSPO *rs6971* polymorphisms (104). The *rs6971* polymorphisms, however, affects *in vivo* [^18^F]GE-180 quantification, revealing lower binding in patients of low-affinity binders compared to the mixed- and high-affinity binders (88). Several studies have compared the binding properties and performance of second and third generations of TSPO tracers. James et al. found that the detection of microglial activation by using [^18^F]GE180 was more sensitive than that by using [^18^F]PBR06 (94). However, Chaney et al. indicated that [^11^C]DPA-713 PET reflects microglial activation with higher accuracy and sensitivity compared to [^18^F]GE-180 in a mouse model of stroke (53). Head-to-head comparative PET study by Zanotti-Fregonara et al. showed a more favourable brain entrance property of [^11^C]PBR28 compared to [^18^F]GE-180 in human (76). [^11^C]ER176 has demonstrated a higher binding potential and smaller variability compared to [^11^C]PK11195, [^11^C]PBR28, and [^11^C]DPA-713 (75, 105, 106). Clinical trial of PET using [^11^C]ER176 for accessing microglia activation in patients with MCI and AD is still ongoing (NCT03744312). Microglial activation assessed by using [^18^F]GE-180 in different amyloidosis, tauopathy rodent models have been reported (47, 50, 53, 89, 90, 92–102) (**Table 1**). López-Picón et al. showed that [^18^F]GE-180 signal reached plateaus at an early stage, while the Aβ load detected by [^11^C]PIB was still increasing in APP23 mice (90). A recent study by Sacher et al. showed an asymmetric pattern (hemispheric predominance) of Aβ load ([^18^F]florbetaben) accompanied by microglial activation ([^18^F]GE-180) in App^NL-G-F^ knock-in mice (96). Increased levels of [^18^F]GE-180 uptake indicative of microglial activation have been reported in patients with AD, semantic dementia, MCI, and four-repeat tauopathy compared to non-demented controls (88–91). Ramakrishnan reported that [^18^F]GE-387 visualised increased uptake in rat of acute inflammation induced by lipopolysaccharides (LPS) injection and demonstrated sufficient brain uptake in non-human primate (104).

The cellular location of the signal is another major concern for TSPO ligands. Two different binding sites on glial and vascular TSPO were reported for several TSPO ligands, e.g., [^11^C]PK11195 (57). Ji et al. reported that polymorphism-insensitive ligand [^18^F]FEBMP (200) yielded a higher contrast to neuroinflammation than [^11^C]PK11195 in PS19 tauopathy mouse model due to its higher glial-TSPO selectivity (**Figures 1F, G** **)** (57, 58). Further studies evaluating the TSPO selectivity and insensitivity to *TSPO* polymorphism of the second and third generations TSPO tracers including [^18^F]GE-180, (S)-[^18^F]GE-387, and [^11^C]ER176 are highly desired.

### Emerging Targets

Given that TSPO is not exclusively expressed in glia, it is thus imperative to search for new imaging biomarkers that can detect neuroinflammation with higher sensitivity and specificity. Promising targets should have almost exclusive expression in microglia or astrocyte and highly specific ligands to enable *in vivo* imaging evaluations (32, 170, 201, 202).

#### Colony-Stimulating Factor 1 Receptor

CSF1R is expressed mainly on microglia and on infiltrating macrophages/monocytes and dendritic cells in the brain (**Figures 1A, B** **)**. CSF1R is important for microglia growth, proliferation, and survival. Two endogenous ligands, the growth factors colony stimulating factor-1 and interleukin-34 (203), have been reported for CSF1R. Upregulation in CSF1R have been reported in response to injury and AD-related neuropathology (204, 205). Horti et al. developed a new CSF1R tracer [^11^C]CPPC and captured increased microglial levels of CSF1R in animal models of acute inflammation induced by LPS injection, encephalomyelitis model of multiple sclerosis, and APPsi with cerebral Aβ pathology (124). A recent study from Zhou et al. compared new CSF1R tracers [^11^C]GW2580 with [^11^C]CPPC in detecting both acute inflammation induced by LPS injection and chronic inflammation in APP^NL-G-F/NL-G-F^ knock-in mice and showed that [^11^C]GW2580 captured changes in CSF1R with higher sensitivity, associated with increased TSPO pattern in the brain (64) (**Figures 1C–E**).

#### Cyclooxygenase-1 and Cyclooxygenase-2

Cyclooxygenase (COX) is an enzyme involved in the production of prostaglandin H2, which is the substrate for molecules including prostaglandins, prostacyclin, and thromboxanes (206). The two isoforms COX-1 and COX-2 are considered to be involved in the neuroinflammation in neurodegenerative diseases including AD. Immunochemical evidence showed that COX-1 and COX-2 are expressed in microglia and neuron in the central nervous system (207). Several tracers for COX-1 and COX-2 have been developed including [^18^F]TMI (131, 208), [^18^F]triacoxib (209), [^11^C]rofecoxib (210), [^11^C]KTP-Me (125, 127, 211), [^11^C]PS13, and [^11^C]MC1 (128, 129) (**Table 1**). Ohnishi et al. and Shukuri et al. reported that [^11^C]KTP-Me harbours an improved brain–barrier entrance and is highly selective for COX-1 (125, 127, 211). PET study with [^11^C]KTP-Me showed an increased brain uptake in AD patients compared to healthy controls and in APPswe (Tg2576) mice compared to wild-type mice (125–127): [^11^C]KTP-Me accumulation was detected in the frontal cortex and hippocampus, in activated microglia surrounding Aβ plaques. Shrestha et al. reported PET imaging of COX-2 ([^11^C]MC1) and COX-1 ([^11^C]PS13) in monkey brain after LPS-induced neuroinflammation and in human peripheral tissue with inflammation and showed specific detection patterns (128, 129).

#### Cannabinoid Receptor Type 2

Cannabinoid receptor type 2 (CB_2_R) are mainly expressed by immune cells including monocytes, macrophages, and microglia in the brain (151, 152) and have low expression levels under physiological conditions (2, 4, 31). Several classes of tracers for CB_2_R have been developed including [^11^C] methoxy-Sch225336 (212), [^11^C]NE40 (154), [^11^C]A-836339, [^18^F]2f (149, 150), [^18^F]JHU94620 (153), [^18^F]RS-126, and [^18^F]RoSMA-18-d6 (151, 152) (**Table 1**). Upregulation of brain CB_2_R expression has been demonstrated in acute inflammation such as LPS-injected model and murine stroke model (151–153) in chronic inflammation senescence-accelerated models (155) and in amyloidosis mouse model associated with Aβ deposits (150). Ahmad et al. reported lower CB_2_R availability in Aβ-positive AD patients compared to healthy controls assessed by PET using [^11^C]NE40 and [^11^C]PIB, respectively. However, no relationship between [^11^C]NE40 and cerebral Aβ load was observed (154).

#### Purinergic P2X7 Receptor and P2Y12 Receptor

The expression of purinergic P2X7 receptor is found upregulated specifically in M1 microglia. P2X7 receptor mediates NLRP3 inflammasome activation, cytokine and chemokine release, T lymphocyte survival and differentiation, transcription factor activation, and cell death (213). Microglia monitors and protects neuronal function through purinergic P2Y12 receptor-dependent junctions (214) linked with neuronal mitochondrial activity. Brain injury-induced changes at somatic junctions triggered P2Y12-receptor-dependent microglial neuroprotective effect, regulating neuronal calcium load and functional connectivity (215, 216). Immunohistochemical staining indicated that the levels of P2Y12 receptor were decreased in the brains derived from patients with multiple sclerosis and AD cases (217). Several P2X7 receptor tracers including [^11^C]GSK1482160 (110, 111), [^11^C]JNJ-47965567 (A-740003) (120), [^18^F]JNJ-64413739 (112, 114), [^11^C]JNJ-54173717 (113), [^11^C]SMW139 (118), and [^18^F]PTTP (218). Janssen et al. showed that [^11^C]SMW139 can detect with high affinity and specificity to the P2X7 receptor by using rAAV3flag-hP2X7R rat model overexpressing human P2X7 receptor (119). Moreover, [^11^C]SMW139 showed higher binding on postmortem brain of AD patients compared to controls by using *in vitro* autoradiography studies, corroborating with immunohistochemical staining results (119). One clinical trial is ongoing using [^11^C]SMW139 for imaging neuroinflammation in Parkinson’s disease [(PRI-PD) 2018-000405-23].

Several P2Y12 receptor probes such as [^11^C]AZD1283, [^11^C]P2Y12R-ant, and [^11^C]5 have been developed and evaluated *in vivo* in animal models (120, 122, 123). Maeda et al. showed a distinct response of P2Y12 receptor to tau and amyloid deposits using P2Y12 receptor tracer [^11^C]AZD1283. The levels of P2Y12 receptor decline in tau-laden region with increased total level of microglia in rTg4510 and PS19 tau mice and increase in APP23 and APP^NL-F/NL-F^ mice (123). However PET imaging using [^11^C]AZD1283 showed no uptake signal in the wild-type mouse brain. Two other tracers [^11^C]P2Y12R-ant and [^11^C]5 have showed sufficient brain uptake and promising results in experimental autoimmune encephalomyelitis model of multiple sclerosis (120) and stroke model for detecting anti-inflammatory microglia (122).

### Astroglia Imaging

#### MAO-B

Irreversible MAO-B inhibitors [^11^C]deuterium-L-deprenyl (DED) have been used in PET imaging studies and demonstrated early astrocytosis in sporadic and autosomal dominant AD patients (61, 156–161, 163) and in amyloidosis mouse models (163, 164). [^18^F]fluorodeprenyl-D_2_ showed favorable kinetic properties with relatively fast washout from non-human primate brain and improved sensitivity for MAO-B imaging (165). However, the technical challenges of irreversible inhibitors such as deprenyl hinder the accurate image analysis. Several reversible-binding inhibitors have been developed in recent years such as [^11^C]Cou (170, 219), [^11^C]SL25.1188 (168), and [^11^C]SMBT-1 (166). Harada et al. showed a specific increased regional retention of [^11^C]SMBT-1 in the cortical and hippocampal regions in patients with AD compared to healthy controls (166).

#### I_2_BS

I_2_BS that locates on both monoamine oxidases A (MAO-A) and B (MAO-B) is another emerging target for astrocytosis imaging (173–175, 220). [¹¹C]FTIMD shows the specific-binging to I₂BS as shown by PET and autoradiography in the monkey brain (183). Wilson et al. demonstrated reactive astroglia detected by using [^11^C]BU99008 PET early in Parkinson’s disease in response to α-synuclein accumulation (174). Recent postmortem binding and autoradiography study by Kumar et al. showed increased level of [^3^H]BU99008 binding in postmortem brain tissue from patients with AD compared to healthy controls (173, 221). Calsolaro et al. recently demonstrated increased cortical astrocytosis assessed by [^11^C]BU99008 with high cerebral Aβ load assessed by [^18^F]florbetaben in patients with MCI and AD (176) (**Figures 2A–C**). Livingston et al. demonstrated that increased astrocytosis assessed by [^11^C]BU99008 in regions of earlier stages with low Aβ loads assessed by [^18^F]florbetaben and reduced astrocytosis in regions of advanced stage with greater Aβ load and atrophy (177). *In vitro* autoradiography and immune-histochemical staining showed the specificity of [^3^H]BU99008 and the colocalization of with glial fibrillary acidic protein staining of astroctyes in brain tissues from patients with AD.

## Discussion

Non-invasive detection of central pathologies is indispensable for understanding the mechanism underlying AD continuum and for facilitating early and differential diagnosis (28, 222–225). TSPO-PET is still the most powerful imaging tool for AD-associated neuroinflammation but is currently facing two challenges. First, a human *TSPO* polymorphism *TSPO rs6971* commonly affects the binding affinities of the second generation tracers to a different extent. Classification with polymorphism enables to correct the variability and bias from different binding affinities, but it raises the threshold for sample size of human subjects. Third-generation tracers have been developed for circumventing this limitation. *In vitro* testing in *post-mortem* human brain tissues have demonstrated the insensitivity of [^11^C]GE-180, [^11^C]GE-387, and [^11^C]ER176 to *TSPO* polymorphism (75, 106, 197). However, recent clinical study with [^11^C]ER176 (105) and [^11^C]GE-180 (88) demonstrated a significant decrease in ligand retention in low-affinity binders, suggesting the necessity of further *in vivo* examination. Second, the heterogenous cellular sources of TSPO PET tracers have been demonstrated in astrocytes, endothelial cells, and vascular smooth muscle cells, in addition to microglia in both patients with AD and animal models (61, 85, 86, 193, 226–229) (**Figures 1A, B** **)**. Although conventional opinions consider microglia as major cellular source of TSPO in the central nervous system, latest study finds vascular TSPO provides major binding sites for TSPO ligands including most widely used [^11^C]PK11195 and [^11^C]PBR28 in normal mouse brains (57). These findings suggest the possibility that changes in TSPO PET signal may be partly due to changes in the levels of vascular TSPO and not purely of glial TSPO. [^18^F]FEBMP and [^11^C]AC-5216 showed relatively selectivity for glial-TSPO compared to other ligands such as [^11^C]PK11195 (200). It remains to be investigated whether the third generation of TSPO tracers shows a portion of vascular TSPO detection similarly. Moreover, further research on next generations of TSPO tracers are needed, with the selection criteria including optimal binding property, insensitivity for *TSPO* polymorphism, and high glial TSPO selectivity.

The role of neuroinflammation in AD pathogenesis is still not fully elucidated. Early clinical studies with first generation tracer [^11^C]PK11195 showed conflicting results in the brains from AD patients. Some studies demonstrated significant increases in [^11^C]PK11195 retention in diseased brain regions in AD (230, 231), which was not observed in some other studies (232, 233). Albrecht et al. recently reported negative associations between regional Aβ and tau PET uptake and CSF inflammatory markers in patients with AD and in non-demented controls and suggested a protective role of neuroinflammation (234). Ewers et al. showed that a higher CSF level of soluble TREM2 is indicative of microglia activation in patients with AD. The CSF level of TREM2 negatively aassociated with the rate of Aβ accumulation assessed by using [^18^F]florbetapir over 2-years follow-up in AD patients (101). Biphasic trajectory with an early increase and a later decline in the level of microglial activation might explain such inconsistency between results from clinical studies (62). The recently reported biphasic trajectory of astrocytosis (177) adds further complexity in the interpretation.

A recent study has showed that microglia is involved in the formation of senile plaque by promoting the diffuse form converting to dense cored form (15). *In vitro* immunohistochemical analysis found that TSPO-positive microglia were surrounded dense cored plaque, not diffuse plaques (235). These results may explain the complex spatial association between TSPO-PET and amyloid-PET signals. [^11^C]PBR28 signal correlated with both tau aggregation and Aβ deposition (55), suggesting distinct dynamic profiles of microglial activation. Collectively, current clinical studies have not provided a consensus on association between TSPO-associated neuroinflammation and AD-pathological changes. Given the different binding sites in glial and vascular TSPO for different tracers, the divergent results using different TSPO-PET tracers are not unexpected. A multitracer imaging paradigm for detecting the regional patterns of Aβ, tau, and microglia activation and astrocytosis is expected to provide better temporal and spatia mapping of disease processes and assessment of immunomodulatory therapeutic interventions in clinical study.

Several promising targets and tracers for neuroinflammation imaging have been reported but not yet been evaluated in AD patients or animal models, such as the ligands for inducible nitric oxide synthase ([^18^F]FBAT), reactive oxygen species ([^18^F]ROStrace [^18^F]ox-ROStrace, [^18^F]dihydromethidine, [^11^C]Ascorbic. [^62^Cu]ATSM, [^11^C]dehydroascorbic acid) (132–137), TREM-1 ([^64^Cu]TREM1-mAb), matrix metalloproteinases ([^18^F]BR-351, [^18^F]BR-420) (144–146), astrocyte metabolism ([^11^C]acetate) (171, 172), I_2_BS([¹⁸F]FEBU) (182), and organic anion-transporting polypeptide 1C1 ([^18^F]2B-SRF101) (184). More preclinical and clinical evidence are required to indicate the utilities of these emerging ligands in *in vivo* imaging. An almost exclusive expression of CSF1R and P2X7 receptor and P2Y12 receptor in microglia have demonstrated their potentials as next-generation imaging targets for microglia activation. Further evaluation of these tracers in amyloidosis and tauopathy models and patients with MCI and AD will potentially facilitate better phenotyping of microglia activation. The association of these emerging targets with AD pathologies, disease progression, and the improvement in the ligand binding properties and analysis methods for PET data require further investigations (236). With the advances in new techniques, e.g., single-cell analysis of neuroinflammatory responses and plasma biomarkers, the link between neuroinflammation PET with other indicators will likely be studied in a more systematic manner.

## Author Contributions

RZ, BJ, and RN wrote the first draft and prepared the figures. All authors contributed to the article and approved the submitted version.

## Funding

RN acknowledged the funding by Helmut Horten Stiftung, Vontobel Stiftung, UZH Entrepreneur Fellowship (reference no. MEDEF-20-021).

## Conflict of Interest

The authors declare that the research was conducted in the absence of any commercial or financial relationships that could be construed as a potential conflict of interest.

## Publisher’s Note

All claims expressed in this article are solely those of the authors and do not necessarily represent those of their affiliated organizations, or those of the publisher, the editors and the reviewers. Any product that may be evaluated in this article, or claim that may be made by its manufacturer, is not guaranteed or endorsed by the publisher.
